# Dental health of patients with X-linked hypophosphatemia: A controlled study

**DOI:** 10.3389/froh.2023.1087761

**Published:** 2023-03-21

**Authors:** Amila Larsson, Tobias Regnstrand, Pia Skott, Outi Mäkitie, Sigridur Björnsdottir, Karin Garming-Legert

**Affiliations:** ^1^ Department of Dental Medicine, Karolinska Institutet, Huddinge, Sweden; ^2^ Public Dental Health Service Stockholm AB, Stockholm, Sweden; ^3^ Department of Pediatric Research, Children's Hospital, Pediatric Research Center, University of Helsinki and Helsinki University Hospital, Helsinki, Finland; ^4^ Department of Clinical Genetics, Clinical Genetics, Karolinska University Hospital, Stockholm, Sweden; ^5^ Department of Molecular Medicine and Surgery, Karolinska Institutet, Stockholm, Sweden; ^6^ Department of Endocrinology, Metabolism and Diabetes, Karolinska University Hospital, Stockholm, Sweden

**Keywords:** dental, endodontic, infections, periodontal, XLH

## Abstract

**Objective:**

The present study compared the dental health of patients with X-linked hypophosphatemia (XLH) with healthy age- and gender-matched controls to increase our knowledge of the impact of XLH on oral health.

**Materials and methods:**

Twenty-two adult patients with XLH in the Stockholm region of Sweden were referred to the Department of Orofacial Medicine at Karolinska Institutet for an extended clinical and radiological examination. Pre-existing radiologic examinations of 44 healthy age- and gender-matched controls were retrieved from the Department of Oral Radiology, at Karolinska Institutet.

**Results:**

The 22 patients with XLH (15 females, median age 38 years, range 20–71; 7 males, median age 49 years, range 24–67) had a significantly higher number of root-filled teeth compared to healthy controls (*p* = .001). In the XLH group, females had significantly better oral health than males, especially concerning endodontic and cariological status (*p's* = .01 and .02, respectively). Periodontal status differed non-significantly between the XLH and control groups.

**Conclusion:**

Patients with XLH had a significantly lower oral health status compared to a healthy population especially concerning endodontic conditions. Male patients with XLH had a higher risk of poor oral health compared to female patients with XLH.

## 1. Introduction

X-linked hypophosphatemia (XLH) is a rare genetic disorder caused by a mutation in the phosphate regulating endopeptidase homolog X-linked (*PHEX*) gene (1). High levels of Fibroblast Growth Factor-23 (FGF23), renal phosphate wasting, and decreased synthesis of active vitamin D are consequences of mutations in *PHEX*. The estimated incidence of XLH in Sweden is 5/100,000 live births (2). Studies from Denmark, Norway, and Japan have found prevalences ranging from 1.7 to 4.8/100,000 children (3–5). Due to its X-chromosomal dominant inheritance pattern, XLH is more prevalent in women than in men. Most cases are familial but *de novo* mutations are also common (6).

XLH mainly affects the skeleton and dentition with the hypophosphatemia causing abnormal mineralization of bone and dentine, rickets, osteomalacia, and short stature (7). XLH can also cause bone and joint pain, muscular fatigue due to hypophosphatemia, bone fractures or pseudo-fractures, and enthesopathy. Lower limb surgery is often needed to correct deformities. Craniofacial malformations occur when the sutures of the skull close prematurely. Hearing loss is also common and can debut in childhood (8).

Impaired mineral quality of dentine and disturbed formation of cementum are some of the reasons contributing to dental issues in patients with XLH. Dental complications such as *spontaneous* periapical infections in apparently intact teeth and periodontal attachment loss are common (9). Higher caries susceptibility, however, is not a known problem associated with XLH (9–12).

When not diagnosed at birth due to a positive family history, symptoms of XLH often appear at a young age as skeletal deformities such as bowed legs and short stature. Owing to its rarity, however, diagnosis and treatment of XLH are often delayed. XLH is usually suspected based on clinical features and a biochemistry panel of low serum phosphate levels, low renal threshold values for reabsorption of phosphate in the urine, and high levels of FGF23; a positive genetic test for a *PHEX* mutation at Xp22.1 is often needed to confirm XLH (1).

The severity of musculoskeletal complications and dental health depends on how early these individuals receive treatment (9, 13–15). Conventional treatment of XLH is based on oral phosphate supplementation and active vitamin D. Doses are highly dependent on age and serum phosphate levels. Risks of conventional treatment include a higher risk of nephrocalcinosis due to an increase in calciuria; another risk is secondary hyperparathyroidism, due to long-term stimulation of the parathyroid cells by phosphate supplementation (16). In children, conventional treatment promotes growth, reduces pain, corrects leg deformities, and substantially improves dental health (15, 17–19).

Conventional treatment in adults improves osteomalacia and dental health and reduces pain; it is thus recommended in symptomatic adult patients with XLH (1). Evidence that enthesopathies or hearing loss improve with conventional treatment in asymptomatic adults is limited. In contrast, evidence for oral health improvements such as reductions in periapical abscesses and periodontitis is substantial (9, 13). Some evidence indicates that longer treatment in adulthood with active vitamin D and oral phosphate has beneficial effects on future dental health and decreases the number of dental abscesses (9, 10, 20).

However, compared with patients who do not have XLH, healing after endodontic treatment in patients with XLH often fails, leading to persisting periapical lesions (21). Periapical lesions in patients with XLH can occur in teeth that were not affected by caries or previous trauma; these lesions are thus at risk of being overlooked during dental examinations.

Dental manifestations in the adult population with XLH remain inadequately characterized. The main purpose of this study was to compare dental health status in an adult Swedish population of patients with XLH with a healthy matched population.

## 2. Materials and methods

### 2.1. Patients and methods

The study involved 22 adult patients diagnosed with XLH at the Department of Endocrinology at Karolinska University Hospital, Solna, and referred to the Department of Dental Medicine at Karolinska Institutet, Stockholm, between October 2020 and May 2021. The investigating dentist (AL) made all clinical and radiological examinations at the Department of Dental Medicine at Karolinska Institutet; a Planmeca Prosensor® HD intra-oral sensor (Planmeca, Planmeca Oy, Helsinki, Finland) and a Planmeca ProMax® were used in the panoramic examinations.

### 2.2. Radiologic examination

The radiologic examination included bite-wing radiographs; a full-mouth periapical radiographic examination for identifying periapical lesions, also in clinically intact teeth; and a panoramic radiograph. All patients had a panoramic radiograph taken on the same day as the clinical and intra-oral x-ray examinations except for one patient, who was examined clinically and had intra-oral radiographs taken 2 weeks later.

One patient was edentulous and exempted from all radiological examinations but the panoramic.

### 2.3. Clinical examination

The clinical examination included registration of manifest caries and a periodontal exam that included measurements of pocket depth, furcation involvement, and tooth mobility. Periodontal diagnoses were made according to the 2017 new classification of periodontal diseases (22). Endodontic status was determined using Roeko Endo-Frost cold spray to check the pulp vitality of all teeth; also, all teeth were inspected for signs of attrition and abrasion. Abnormalities in the oral mucosa were noted, except for those compatible with anatomic variations. Clinical diagnoses were established according to the International Classification of Diseases (23). All patients were screened for TMD.

The control group comprised 44 healthy age- and gender-matched individuals with pre-existing radiologic data from radiology exams at the Department of Dental Medicine at Karolinska Institutet (Table 1). Inclusion criteria for the control group were radiologic exams with a panoramic radiograph and a radiologic full-mouth status, and no signs of XLH. Two controls were matched according to age and gender for each patient in the XLH group.

**Table 1:** Patient characteristics of 66 analysed patients including both XLH and control group, aged 20–72.

Characteristics	XLH	Control
Numbers	22	44
Age, median (range)	38 (20–71)	39 (25–72)
Sex (M/F)	7/15	14/30
Other diseases (yes/no)	7^a^/15	10^b^/34
Symptom debut, age, median (IQR)	1 (0–29)	
Diagnosis, age, median (IQR)	1 (0–37)	
Family members affected by XLH (yes/no)	17/5	
Medication (conventional^c^/burosumab)	21/0	
Medication compliance/non-compliance	18/3	
Surgery of the femur (yes/no)	16/5	
Hearing loss due to XLH (yes/no)	4/22	

IQR, Interquartile range.

^a^ Other medical conditions among patients with XLH: hypertension, gout, hypothyroidism, aortic stenosis, attention deficient hyperactive disorder (ADHD), depression.

^b^ Other medical conditions among controls: hypertension, asthma, hypothyroidism, one patient with muscular sclerosis (MS) at an early stage.

^c^ Traditional medication: phosphate, active D-vitamin.

The same dentist who examined the patients clinically (AL) interpreted the radiologic data in Planmeca Romexis®. The blinded interpretation of the radiologic data in the XLH group started 8 weeks after the dental exam. Before interpretation of other data, inter-observer reliability between a resident in dentomaxillofacial radiology at the Department of Dental Medicine, Karolinska Institutet (TR), supervised by a specialist in dentomaxillofacial radiology, and the dentist (AL) was determined by calibrating their scores for 20 pre-existing full radiologic statuses. All radiologic data were interpreted in a dark room with low ambient backlighting on an EIZO Radi Force RX 250 screen, a self-calibrating LCD screen. After at least 2 weeks, a new blinded interpretation of the XLH radiologic data was done to check intra-observer reliability. The same procedures used in the XLH group for interpreting radiologic data were used in the control group.

After the blinded interpretations of the radiologic data in the two groups, the findings of the radiologist and the dentist were compared to determine inter-observer reliability. The final radiologic results are based on the consensus between the radiologist and the dentist.

Caries, alveolar bone level, angular bone defects, number of existing root fillings, apical lesions, root resorptions, and number and location of lost teeth were evaluated. Manifest and secondary caries weredefined as lesions extending into the dentine. Alveolar bone loss was measured in millimetres from the cemento-enamel junction to the marginal bone and recorded as the average between the largest and smallest measured loss. The number of angular bone defects exceeding a depth of 3 mm and a horizontal width of 2 mm was recorded (24). The number and location of root fillings were recorded. A root filling was defined as a tooth with a gutta-percha filling in the root. Periapical lesions were recorded as being (a) in root-filled teeth or (b) in totally intact teeth. Root resorptions (internal and external) were recorded. Besides number, the location of lost teeth was noted as (a) anterior maxillary (incisors and canines), (b) anterior mandibular (incisors and canines), (c) lateral maxillary (premolars and molars), and (d) lateral mandibular (premolars and molars). The third molars were excluded.

### 2.4. Statistical analysis

Intra- and inter-observer reliability were analysed using the *F*-test and are reported as average values. Between-group differences in radiologic variables were tested using the two-sided Wilcoxon signed-rank test with continuity correction, where values for the patients were compared to the mean of the values for the two controls. Differences between genders were calculated using the two-sample Wilcoxon rank-sum test (i.e., the Mann-Whitney *U* test). When possible, medians of the differences and corresponding 95% confidence intervals (95% CI: min–max) were calculated for all variables with a *p*-value <.05; this was not possible, however, for the variables *caries* and *missing anterior teeth in the lower jaw* due to the distribution of the values. For the variable *lost anterior mandibular teeth,* all controls had a value of 0. The ranges of the 95% CIs are based on normal approximations. Differences were considered significant at *p* < .05. All other data are shown as medians (min, max).

## 3. Results

### 3.1. Cohort characteristics

Patients were aged 20–71 years. A diagnosis of XLH was based on a genetic test, a positive family history, or biologic markers such as low levels of phosphate or high levels of FGF23. Most patients (81%) had a positive family history of XLH and had been diagnosed in early childhood (Table 1). All patients began treatment at the time of diagnosis and received the conventional treatment of oral phosphate and active vitamin D; no patient was receiving burosumab. Most patients (86%) reported good compliance with medication from the start of treatment. Seven of 22 patients had other diagnoses as well: hypertension, gout, hypothyroidism, aortic stenosis, ADHD, and depression. Median age of the 7 male patients was 49 years (range 24–67) and of the 15 female patients, 38 years (range 20–71).

### 3.2. Clinical status

One of the 22 patients with XLH was edentulous after tooth loss due to recurrent apical infections and had well-functioning full dentures. Of the remaining patients with XLH, the clinical exam found that 14% (3/21) had manifest and secondary caries lesions.

Periodontally, 50% (10/21) had gingivitis, 19% (4/21) had periodontitis stage II grade B, and 9.5% (2/21), periodontitis stage III grade C. No periodontal pathology was found in nearly one-fifth (19%, 4/21) of the patients with XLH. However, half (11/21) of the patients with XLH exhibited level 1 tooth mobility with mobility in half of these (6/11) concentrated in the anterior teeth of the mandible, regardless of periodontal pathology (absence of bleeding pockets measuring >4 mm) or traumatic occlusion. In 24% (5/21) mobility occurred mainly in the lateral regions of the jaws. In those cases, the anterior teeth in the mandible were lost. Mobility grades 2–3 in 24% (5/21) of the patients occurred mainly in teeth in the anterior mandibular region, regardless of periodontal pathology or traumatic occlusion.

The clinical exam of the XLH group included pulp vitality measurements of non-root-filled teeth in 76% (16/21) of the patients. No signs of oral pathology were detected in the mucosa. One patient reported TMJ-related problems, and 62% (13/21) of the patients had a night guard. No correlation between use of a night guard and tooth mobility was observed.

### 3.3. Radiologic status

#### 3.3.1. Caries prevalence

Radiograph assessment found manifest caries lesions to be significantly more common in the control group [1.00 (0, 9.00)] compared to the XLH group [0 (0, 9.00); *p *= .03]. The number of manifest caries lesions was also significantly higher among men with XLH [1.00 (0, 3.00)] compared to women with XLH [0 (0, 2.00); *p* = .02]. No significant differences between genders in number of manifest caries lesions were found for the control group.

#### 3.3.2. Periodontal status

No significant differences in alveolar bone level (*p* = .58) or number of angular bone defects (*p* = .92) was found between the groups.

#### 3.3.3. Endodontic status

**Figure 1:**
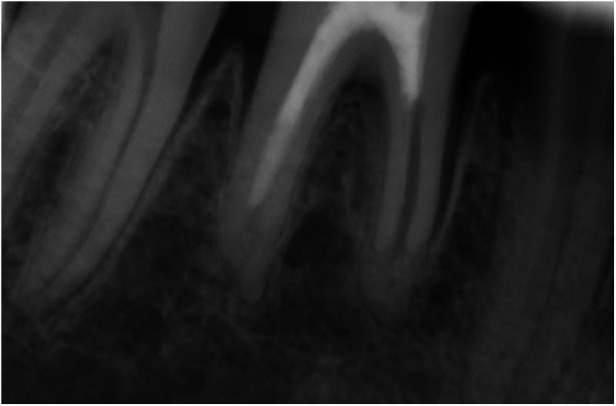
Intra-oral radiograph of a patient with X-linked hypophosphatemia (XLH); note signs of atypical widened periodontal gaps with a continuous lamina dura, preserved marginal bone, and signs of root resorption.

**Figure 2:**
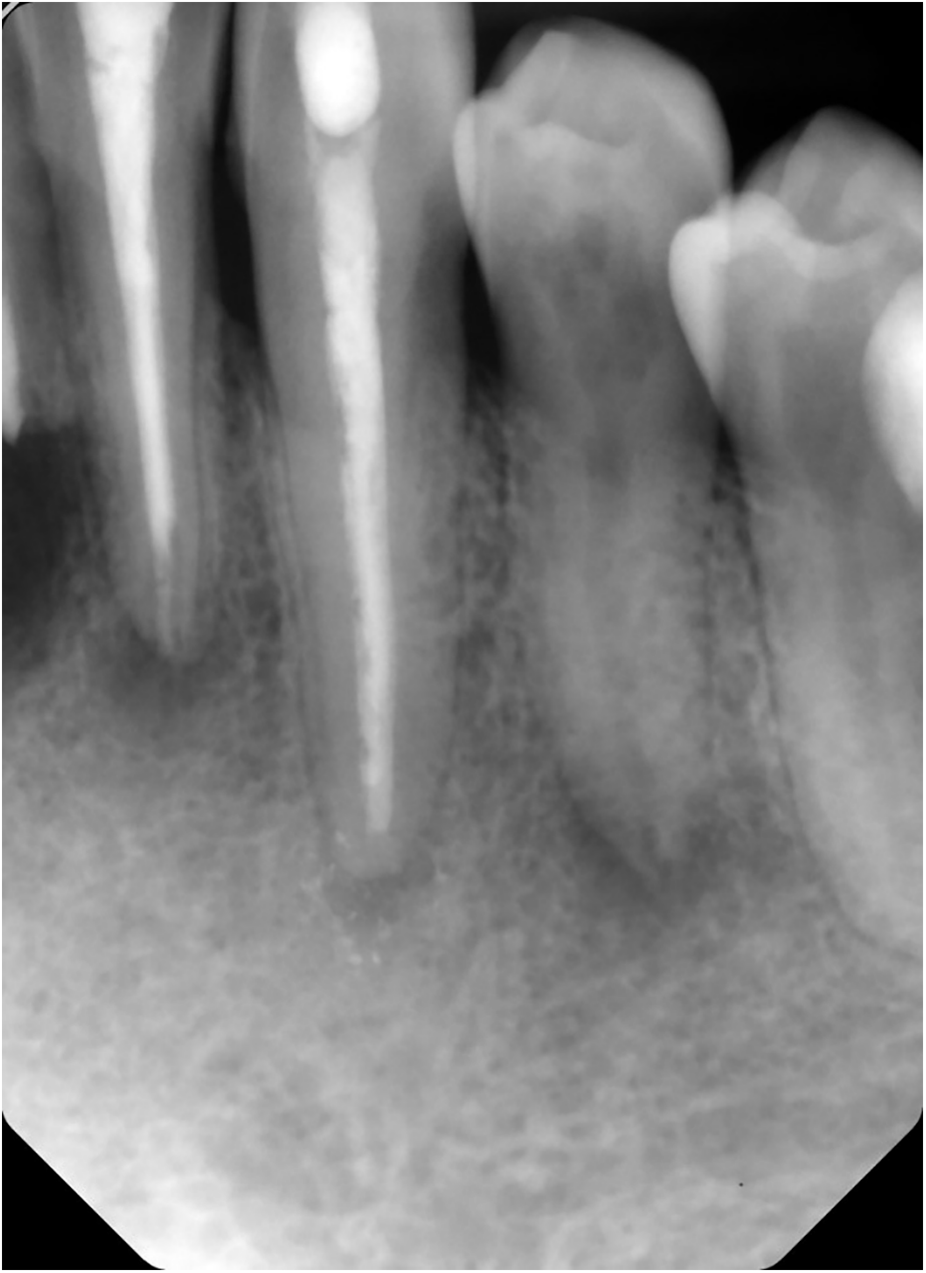
Intra-oral radiograph of a patient with X-linked hypophosphatemia (XLH); note periapical radiolucency on an intact mandibular premolar (25), indicating periapical infection.

**Figure 3:**
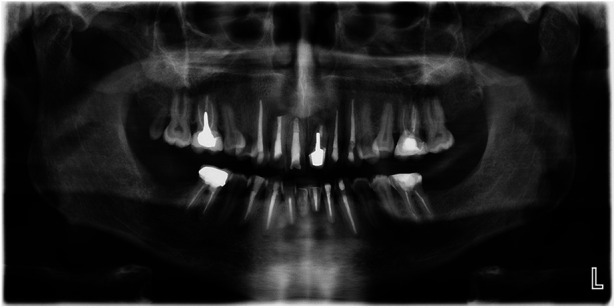
Orthopantomogram of a 46-year-old male patient with X-linked hypophosphatemia (XLH) and numerous root-filled teeth.

**Figure 4:**
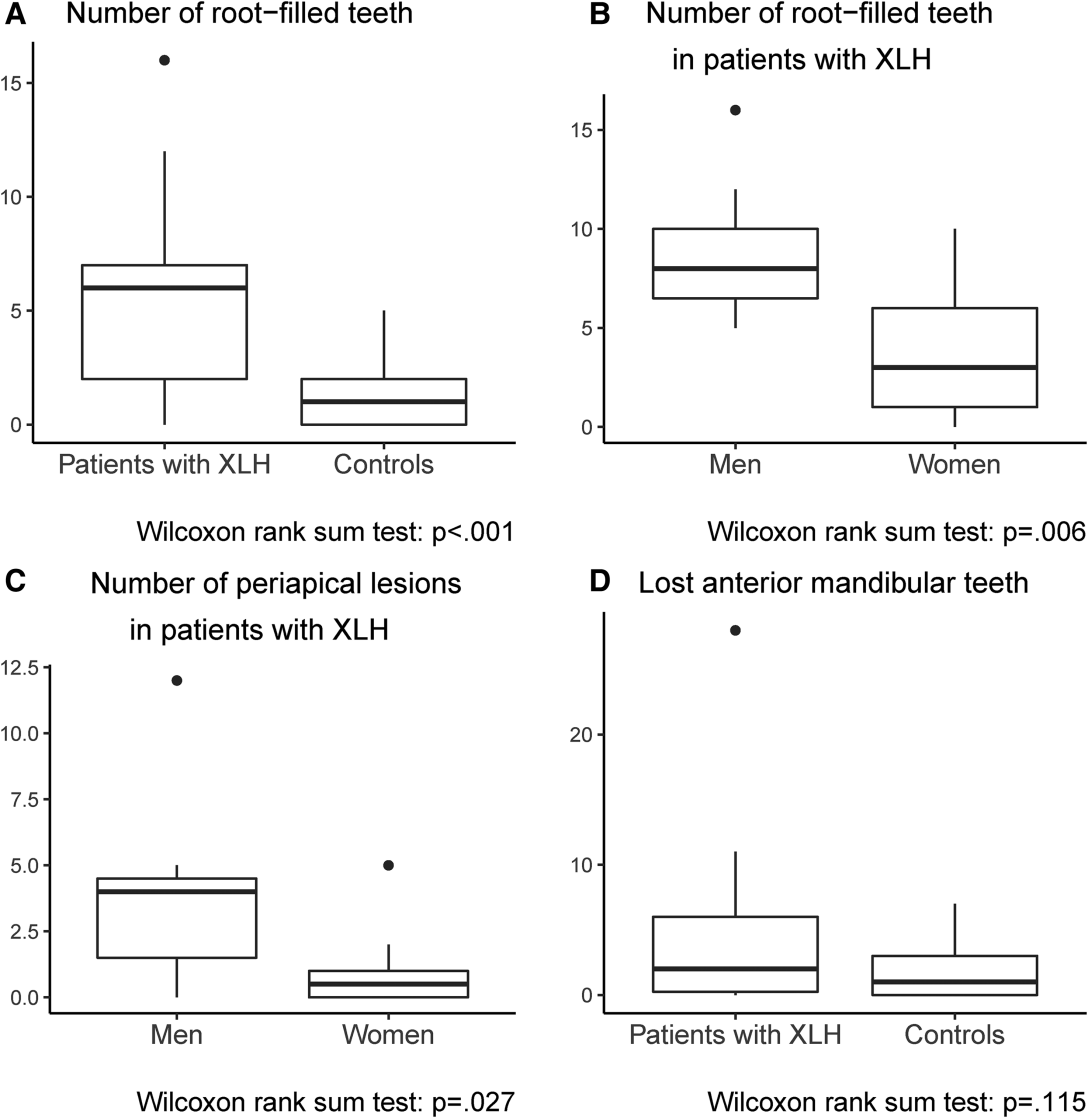
Boxplots showing significant differences in endodontic status between the two groups [the X-linked hypophosphatemia (XLH) group and the age- and gender-matched control group] and between men and women in the XLH group.

In the XLH group, 5 of the 21 dentulous patients had periapical infections on teeth with no other diagnoses such as caries, visible cracks, or previous trauma (Figure 1). Root resorptions associated with root-filled teeth occurred in 6 of the 21 patients (Figure 2). Compared to the control group [1.00 (0, 5.00)], root-filled teeth were significantly more common in the XLH group [6.00 (0, 16.0); *p* = .001] with a median difference of 3.95 (95% CI: 2.00–5.75; Table 2; Figures 3, 4) between the cases and their matched controls. The number of root-filled teeth was significantly higher in the XLH group compared to the controls in most regions of the dental arch: The anterior maxillae (median difference 2.00; 95% CI: 1.25–3.50; *p* = .001), the anterior mandible (median difference 1.75; 95% CI: 1.00–3.00; *p* = .007), and the lateral regions of the maxillae (median difference 1.50; 95% CI: 1.00–2.00; *p* = .003; Table 2). No significant differences were seen in the lateral regions of the mandible for root-filled teeth. Similarly, no differences in numbers of periapical infections were found between the groups (*p* = .05), but we did observe a significant difference between men [4.00 (0, 12.0)] and women [0.50 (0, 5.00)] with XLH (*p* = .03) with a median difference of 2.82 (95% CI: 0.00–4.00; Figure 4). The number of root-filled teeth was also significantly higher in men [8.00 (5.00, 16.00)] than in women [3.00 (0, 10.00)], (*p* = .01) with XLH, with a median difference between the genders of 5.00 (95% CI: 2.00–8.00; Figure 4). This difference occurred in all regions of the jaws but was especially high in the lateral regions of the mandible (*p* = .001).

**Table 2:** Periapical and endodontic status in XLH-group and control group on a tooth level in different localisations in the jaw.

Status	XLH	Control
*Root-filled teeth*		
Mean (SD)	5.29 (4.17)	1.30 (1.44)
Median (min, max)	6 (0, 16)	1 (0, 5)
Anterior maxillary teeth
Mean (SD)	1.67 (1.74)	0.114 (0.493)
Median (min, max)	1 (0, 6)	0 (0, 3)
Anterior mandibular teeth
Mean (SD)	1.24 (1.55)	0.0682 (0.452)
Median (min, max)	1 (0, 5)	0 (0, 3)
Lateral maxillary teeth
Mean (SD)	1.52 (1.33)	0.364 (0.750)
Median (min, max)	2 (0, 4)	0 (0, 4)
Lateral mandibular teeth
Mean (SD)	0.857 (1.11)	0.750 (1.06)
Median (min, max)	1 (0, 4)	0 (0, 4)
*Apical periodontitis*
Mean (SD)	1.95 (2.85)	0.477 (0.821)
Median (min, max)	1 (0, 12)	0 (0, 3)
*Apical periodontitis in root-filled teeth*
Mean (SD)	1.38 (2.29)	0.250 (0.534)
Median (min, max)	1 (0, 10)	0 (0, 2)
*Apical periodontitis in intact teeth*
Mean (SD)	0.524 (1.12)	0 (0)
Median (min, max)	0 (0, 4)	0 (0, 0)

Five of 21 patients showed signs of rather *atypical widened periodontal gaps* (Figure 2). These gaps were not classed as angular bone defects since the horizontal widths were less than 2 mm and surrounded by intact bone; neither could the gaps be classed as typically widened periodontal gaps due to the appearance of a continuous lamina dura.

Overall, the difference in lost teeth between the XLH and control groups was not significant (*p* = .05). In the anterior mandible, however, patients with XLH had a significantly higher number of lost teeth [0 (0, 6.00)] compared to controls [0 (0, 0); *p* = .01; Figure 4]. This was significantly more common in men [3.00 (0, 6.00)] than in women with XLH [0, (0, 6.00); *p* = .04] with a median gender difference of 5.00 (95% CI: 0.0–4.00), but this did not occur in other regions of the jaw.

### 3.4. Intra- and inter-observer reliability

Intra- and Inter-observer reliabilities for radiologic status were interpreted according to the guidelines of Koo and Li (26): intraclass correlation coefficients (ICCs) below 0.50 were defined as poor, between 0.50 and 0.75 as moderate, between 0.75 and 0.90 as good, and above 0.90 as excellent.

Intra-observer reliability was good or excellent for all radiologic parameters except for measures regarding angular bone defects (operator A: ICC, 0.167; and operator B: ICC, 0.333) and measures regarding apical infections in intact teeth (B: ICC, 0.211; and A: ICC, 0.995). Inter-observer reliability was good or excellent as well for almost all parameters except caries prevalence (average ICC, 0.075), which was poor. Moderate inter-observer reliability, however, was observed for apical infections in intact teeth (average ICC, 0.680) and angular bone defects (average ICC, 0.500).

## 4. Discussion

The present study observed a profoundly compromised dental health in adult patients with XLH compared to healthy controls. This was mainly due to higher numbers of endodontically treated teeth, periapical infections, and missing teeth. To our knowledge, this is the first case-control study in adult patients with XLH and healthy age- and gender-matched controls. Several reasons have been proposed for the high susceptibility of patients with XLH to periapical infections, such as abscesses and fistula building: pulp chamber anatomy, which is larger than in the controls; an impaired quality of dentine; and structural differences in the enamel that allow bacteria that are conducive to pathologic periapical processes to harbour (10, 27–30). In our study, 5 of 21 patients had periapical infections on intact teeth that were not related to previous trauma, showed no signs of visible enamel infractions, and had no carious lesions. This underlines the need of regular extended radiographic exams in this patient group for detecting signs of periapical pathology in apparently intact teeth.

Despite the X-chromosomal inheritance pattern, no major sex differences in XLH severity are usually seen (31). However, our findings suggest that dental morbidity in men with XLH is more severe than in women. These findings are similar to Baroncelli et al. (32), who reported that all male patients with XLH had abscesses in teeth unaffected by trauma or decay, while only a subgroup of females suffered from abscesses. Although XLH is an X-chromosomal disease, there is no clear evidence for why the disease would be more severe in hemizygous males compared to heterozygous females. In contrast, our findings indicate a possible sex difference in the severity of dental consequences of XLH, possibly due to the X-chromosomal nature of the disease. It is also possible that differing behavioural patterns could explain this. In our study, male patients with XLH had visited the dentist more frequently for acute dental visits in the preceding year than female patients, which indicates a higher need for dental care.

In our cohort, gender differences occurred in the location of the affected teeth. Significantly more endodontically treated teeth were located in the lateral regions of the mandible in women with XLH compared to men. Differences also occurred between the patient and control groups. Incisors and canines in the anterior mandible were significantly more often endodontically treated than teeth in the lateral regions of the jaw in patients with XLH. This agrees with Andersen et al. (21), who found that endodontic complications occurred most frequently in the incisors and canines in young ages, with premolars and molars becoming increasingly affected with age. In the anterior region of the mandible, teeth are more prone to both substance loss due to attrition as well as attachment loss due to less bone volume. These circumstances may also explain why the XLH cohort in our study experienced loss of mandibular incisors to a significantly higher degree than of teeth in other regions of the jaw.

Caries prevalence among patients with XLH in the present study was rather low, in line with other studies (9–12). When compared with the controls, the lower caries prevalence in the XLH group was significant, perhaps due to a heightened awareness of the importance of oral health and the necessity of prophylactic measures. However, the control group was chosen from a group of healthy individuals seeking dental care and with indications for needing a full-mouth radiologic exam.

The clinical examination found manifest caries in 3 of the 21 dentulous patients with XLH. However, after examiner calibration, evaluation of the intra-oral radiographs found that 10 of these patients had manifest caries. The significance of this difference testifies to a need for radiographs in this patient group when assessing approximal caries.

In contrast to Biosse Duplan et al., 2017 (9), we found that the prevalence of periodontitis was rather low in our cohort of patients with XLH, with findings of periodontitis in only 6 of the 21 dentulous individuals, of which 2 had a more severe form. Compared with the healthy control group, there were no significant differences in general bone level or number of angular bone defects. Half of the XLH group had some tooth mobility at level 1–2. The mandibular incisors exhibited mobility in 5 of these patients, while another 5 with missing mandibular incisors exhibited mobility of the premolars and molars, indicating that the impact of XLH on their dentition was more advanced. In 3 of the 5 patients with mobility at the mandibular incisors, mobility was grade 2–3. The absence of periodontal pathology or traumatic occlusion in these patients indicates that mobility is due to the impact of XLH on cementum thickness with subsequent impairment of the ligament attachment. A search of the literature revealed no earlier studies showing *atypical widened periodontal gaps* in patients with XLH due to preservation of the lamina dura; this contrasts with the widened periodontal gaps typically seen in pathological periapical processes in patients without XLH. One could speculate that these gaps in patients with XLH are a result of the impact of XLH on cementum thickness since we found no other correlation between periodontal pathology or mobility and these gaps.

On a cellular stage, the teeth of patients with untreated XLH and of patients whose treatment started late or whose treatment compliance was poor exhibited distinct acellular cementum hypoplasia compared to healthy controls; these patients with XLH had more extensive and more severe periodontitis (9). To the best of our knowledge, no previous studies on patients with XLH have assessed tooth mobility in the teeth that were not periodontally affected by loss of general bone level.

To improve periodontal health in patients with XLH, treatment with phosphate and vitamin D should begin as soon as possible to prevent onset or further development of periodontitis (9). In our study, all but one patient was receiving medical treatment in adulthood, and compliance was high, which could explain the rather good periodontal status in this cohort. Further, we found no correlation between early onset of medical treatment with phosphate and vitamin D and dental health; almost all individuals in our cohort began treatment in early childhood, either during infancy or by 4–6 years of age.

None of the patients in our study received treatment with burosumab. Burosumab is a human monoclonal antibody neutralizing the levels of FGF23. Data have shown that burosumab is more effective than conventional therapy in improving rickets, growth, lower limb deformity, and mobility in children with XLH (33). There is evidence that suggests that treatment with burosumab in children with XLH reduces the number of dental abscesses (25, 34). However, further research is needed on the effects of burosumab on dental health in adult patients with XLH.

The present study compared the dental health in a cohort of patients with XLH with an age- and gender-matched control group. Inter- and intra-observer reliability were assessed for the two examiners who analysed the radiologic exams; the same clinician performed all clinical exams of the XLH group. The blinded interpretation of the radiologic data of the XLH group beginning 8 weeks after the clinical exam minimised the risk that the clinical examiner might recall the clinical status of a patient during interpretation. Limitations of the present study include lack of information on previous dental care, especially on periapical pathology, over time in patients with XLH. The number of patients was limited due to the rarity of the diagnosis.

### 4.1. Conclusion

The present study found that oral health was lower in patients with XLH compared to a healthy population, especially regarding apical pathology on clinically intact teeth. Furthermore, gender differences in the oral health of patients with XLH highlight the need for individual risk analyses during treatment.

## Data Availability

The original contributions presented in the study are included in the article/Supplementary Material, further inquiries can be directed to the corresponding author.
